# The Ischial Spine in Developmental Hip Dysplasia: Unraveling the Role of Acetabular Retroversion in Periacetabular Osteotomy

**DOI:** 10.1155/2020/1826952

**Published:** 2020-08-18

**Authors:** Gerard El-Hajj, Hicham Abdel-Nour, Rami Ayoubi, Joseph Maalouly, Fouad Jabbour, Raja Ashou, Alexandre Nehme

**Affiliations:** ^1^Department of Radiology, Saint George Hospital University Medical Center, University of Balamand, P.O. Box 166378, Achrafieh, Beirut 1100 2807, Lebanon; ^2^Department of Orthopedic Surgery and Traumatology, Saint George Hospital University Medical Center, University of Balamand, P.O. Box 166378, Achrafieh, Beirut 1100 2807, Lebanon

## Abstract

**Purpose:**

Radiological diagnosis of acetabular retroversion (AR) is based on the presence of the crossover sign (COS), the posterior wall sign (PWS), and the prominence of the ischial spine sign (PRISS). The primary purpose of the study is to analyze the clinical significance of the PRISS in a sample of dysplastic hips requiring periacetabular osteotomy (PAO) and evaluate retroversion in symptomatic hip dysplasia.

**Methods:**

In a previous paper, we reported the classic coxometric measurements of 178 patients with symptomatic hip dysplasia undergoing PAO where retroversion was noted in 42% of the cases and was not found to be a major factor in the appearance of symptoms. In the current study, we have added the retroversion signs PRISS and PWS to our analysis. Among the retroverted dysplastic hips, we studied the association of the PRISS with the hips requiring PAO. We also defined the ischial spine index (ISI) and studied its relationship to the coxometric measurements and AR.

**Results:**

In hips with AR, the operated hips were significantly associated with the PRISS compared to the nonoperated ones (*χ*^2^ = 4.847). Additionally, the ISI was able to classify acetabular version (anteverted, neutral, and retroverted acetabula). A direct correlation between the ISI and the retroversion index (RI) was found, and the highest degree of retroversion was found when the 3 signs of acetabular retroversion were concomitantly present (RI = 33.6%).

**Conclusion:**

The PRISS, a radiographic sign reflecting AR, was found to be significantly associated with dysplastic hips requiring PAO where AR was previously not considered a factor in the manifestation of symptoms and subsequent requirement for surgery. Moreover, the PRISS can also serve as an adequate radiographic sign for estimating acetabular version on pelvic radiographs.

## 1. Introduction

Subtle variations in normal anatomy of the hip joint labeled by Ganz et al. as CAM and pincer-type impingement can cause a premature contact between the head-neck junction and the anterior wall of the acetabulum leading to early hip osteoarthritis (OA). This was later confirmed by Tanzer et al., and a clear relationship between FAI and early OA was established [1–3]. Reynold defined the crossover sign (COS) and the posterior wall sign (PWS) as the radiological parameters to detect acetabular retroversion on a typical AP pelvic radiograph [4]. The COS is positive in all retroversion cases, whereas the PWS is positive only in hips with deficient posterior wall. Jamali et al. confirmed that the presence of a positive crossover sign is a highly reliable indicator of acetabular retroversion with anatomic correlations performed on 43 cadavers [5]. However, despite being good radiological indicators, the COS and PWS both rely on difficult visualization of the acetabular walls [6–8].

As a result, the prominence of the ischial spine (PRIS) inside the pelvic brim emerged as a more reproducible sign reflecting acetabular retroversion because it is easier to detect. It was first described by Kalberer et al. in 2008, with excellent sensitivity and a positive predictive value [9]. It has been shown to have high interobserver and intraobserver reliability among orthopedic surgeons and radiologists [10]. This sign has been studied in a series of retroverted hips including mostly normally covered hips but never on a series including only patients operated for unilateral or bilateral hip dysplasia.

In fact, in patients with hip dysplasia, whether unilateral or bilateral, the dysplastic retroverted sockets constitute a specific subgroup (42%) [11]. The surgical decision of performing a periacetabular osteotomy (PAO) in this subgroup is dictated by the amount of dysplasia and not by retroversion [12].

However, at the time of the study, the ischial spine was not a well-established sign of retroversion. In our study, we found that the addition of the sign can be used to uncover potential morphological associations reflected by the ischial spine.

Retroversion in the setting of dysplasia does not seem to produce impingement even though a COS is present. In fact, there is global insufficient development of the anterior and posterior walls associated with a steeper inclination of the neck in the setting of increased valgus, which makes a premature contact between both nearly inexistent.

Moreover, it is paramount to diagnose retroversion in those hips in order to be able to correctly orient the osteotomized fragment and thus avoid postoperative impingement. Because when corrected, those hips becoming normally covered but still retroverted, will have the distance of the anterior wall to the head-neck junction shortened, and hence will become symptomatic [13–15].

Therefore, our aim is to study the variation of the PRIS sign (PRISS) in a series of patients undergoing PAOs to correct unilateral or bilateral dysplasia, according to acetabular version, and to the presence (requiring PAO) or absence (conservative treatment) of symptoms in dysplasia.

## 2. Materials and Methods

A total of 227 patients underwent PAO between 1995 and December 2003. Patients who were asymptomatic, even when presenting with radiological signs of congenital dysplasia, were treated conservatively. Only symptomatic patients who were experiencing pain secondary to their congenital hip dysplasia underwent surgeries; 204 patients underwent unilateral PAO, and 23 patients underwent bilateral two-staged PAO.

The following inclusion criteria were used for reviewing the preoperative radiographs:

1. The anterior and posterior walls, the bearing surface, as well as the external edge of the acetabulum were well defined on the radiographs.
2. The symmetries of the iliac wings and the obturator foramens were used to check for neutral rotation.
3. Coccyx to pubic symphysis distance of less than 2 cm was measured to have neutral tilts of the pelvis.
4. Percentage of femoral head coverage was measured on hips in neutral abduction.
5. A Lequesne false profile radiograph was obtained for each patient.

**Figure 1:**
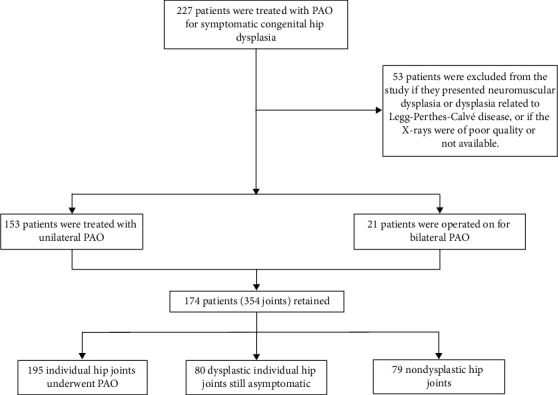
Population with selection criteria.

Radiographs that did not meet the inclusion criteria were excluded from this study. Patients with a diagnosis of neuromuscular dysplasia or Legg-Perthes-Calvé disease were also omitted. The remaining number of patients was 174 (348 hips), with a mean age of 30 years (range, 15–56 years; SD = 10.5), 137 were females (79%) and 37 were males (21%). The selection and total patients are shown in Figure 1.

### 2.1. Radiographic Hip Parameters

**Figure 2:**
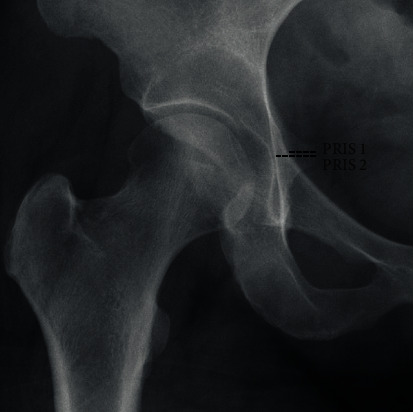
Pelvic AP radiograph showing PRIS 1 and PRIS 2 measurements.

1. Prominence of the ischial spine (PRIS) is an alternate radiographic sign for acetabular retroversion because in these hips, the whole hemipelvis is rotated. PRIS 1 measured the ischial spine protruding into pelvic inlet, and PRIS 2 measured the entire ischial spine extending to the ilioischial line (Figure 2). If the ischial spine extends beyond the pelvic brim, it is considered a positive sign (PRIS 1 > 0).
2. Ischial spine index (ISI), newly described ratio of PRIS 1 over PRIS 2, which accounts for the percentage of the ischial spine protruding into the pelvic inlet.
3. Lateral center-edge (Wiberg's) angle, measured by a vertical line and a line connecting the femoral head center with the lateral edge of the acetabulum. Normal LCE angles ranges from 20° to 40°. Angles below 20° indicate hip dysplasia.
4. Vertical-center-anterior edge (VCA) angle, formed by intersection of a vertical line through the center of the femoral head and a line extending through the center of the femoral head to the anterior sourcil. It measures anterior dysplasia on the false profile view and is an indicator of the degree of femoral head anterior coverage. Normal values range from 20 to 50 degrees.
5. Tönnis angle, formed between a horizontal line and a line extending from the medial to lateral edges of the sourcil. Acetabula having a Tönnis angle of 0°–10° are considered normal, whereas those having an angle of >10° or <0° are considered to have increased and decreased inclination, respectively. Acetabula with increased Tönnis angles are subject to structural instability, whereas those with decreased Tönnis angles are at risk for pincer-type femoroacetabular impingement.
6. The index of extrusion of the femoral head measured as the lateral part of the femoral head not covered by the acetabulum divided by the total width of the head. Values under 25% are usually indicators of an adequately covered femoral head.
7. Acetabular index depth to width: it is the depth of central portion of acetabulum divided by the width of acetabular opening.
8. Acetabular orientation was assessed using the crossover sign.
9. Crossover distance, distance between the superolateral edge of the acetabulum and the crossover sign.

### 2.2. Statistical Analysis

To compare the coxometric measurements according to the presence or absence of the ischial spine, a *t*-test was used to analyze the difference between the 2 groups. A Pearson product-moment correlation coefficient was computed to assess the relationship between length of the ischial spine and the following parameters: retroversion index and crossover distance. The following correlation scale was taken: 0.00–0.19 “very weak,” 0.20–0.39 “weak,” 0.40–0.59 “moderate,” 0.60–0.79 “strong,” and 0.80–1.0 “very strong.” One-way analysis of variance (ANOVA) was used to compare the 3 acetabular version groups. The chi-square independence test was used to study the association between the signs and the surgical hips.

### 2.3. Interobserver Reproducibility

Interobserver reproducibility of ischial spine measurements was evaluated by 2 different radiologists in a subset of 100 hips using a two-way, mixed, consistency single-measures intraclass correlation coefficient (ICC). ICC values greater than 0.80 indicate excellent reliability, 0.61–0.80 substantial reliability, 0.41–0.60 moderate reliability, 0.21–0.40 fair reliability, and <0.20 poor reliability [16]. The ICC showed excellent reliability for measurements of PRIS 1 (ICC ¼ 0.823, 95% confidence interval (CI) 0.776–0.876).

## 3. Results and Discussion

### 3.1. Analysis of the Length of the Ischial Spine

When classified according to acetabular version (anteverted, neutral, and retroverted), there was a statistically significant difference in the ISI between groups as determined by one-way ANOVA (*F* (2,183) = 33.665, *P* < 0.001). A Tukey post hoc test revealed that the ISI was statistically significantly lower in the anteverted (5.64 ± 13.08%, *P* < 0.001) and neutral (21.33 ± 24.68%, *P* < 0.0001) acetabula compared to the retroverted group (34.16 ± 24.83%, *P* < 0.001). The demographics and other radiographic parameters are shown in Table 1.

Furthermore, we found a good positive correlation with the crossover distance (Pearson's *r* = 0.612, *P* < 0.0001) and a moderate positive correlation with the retroversion index (Pearson's *r* = 0.416, *P*=0.003). As for the PRISS 2, we only found significant correlations with the crossover distance (*r* = 0.466) (Table 2).

### 3.2. Coxometric Measurements according to the Presence of the PRISS (Ischial Spine as a Positive or Negative Sign)

When compared according to the PRISS, hips with the positive sign were significantly associated with greater crossover distance compared to those without the PRISS (Table 3).

### 3.3. Validity of the PRISS in Determining Acetabular Version

**Figure 3:**
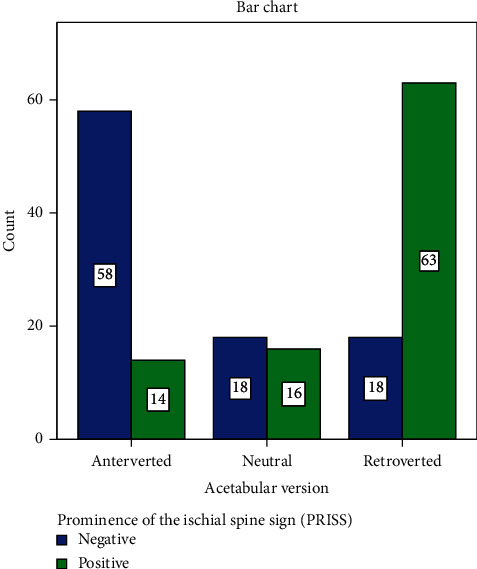
Distribution of the PRISS according to acetabular version (*χ*^2^ (2, *N* = 184) = 52.03, Cramer's *V* = 0.527, *P* < 0.001).

Figure 3 shows the association between the PRISS and acetabular version in hips requiring PAO (*χ*^2^ (2, *N* = 184) = 52.03, Cramer's *V* = 0.527, *P* < 0.001). The proportion of the PRISS in the groups was gradually increasing moving from anteverted, neutral, to retroverted.

Table 4 shows the acetabular retroversion index in different combination of radiographic markers. RI (corresponding to the amount of acetabulum that is retroverted) was found to be highest when all the signs are positive and lowest when all are negative.

**Figure 4:**
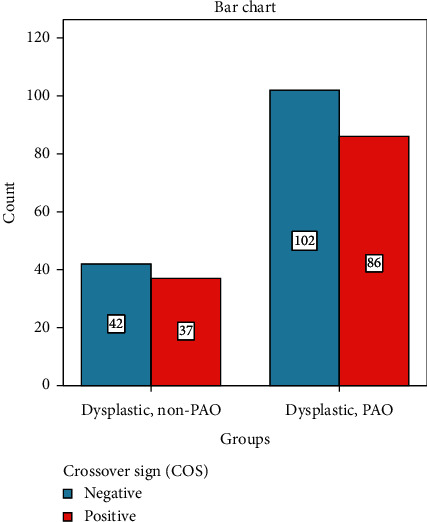
The ischial spine sign in retroverted dysplastic hips (PAO vs. non-PAO); *χ*^2^ = 4.847, *P*=0.027.

**Figure 5:**
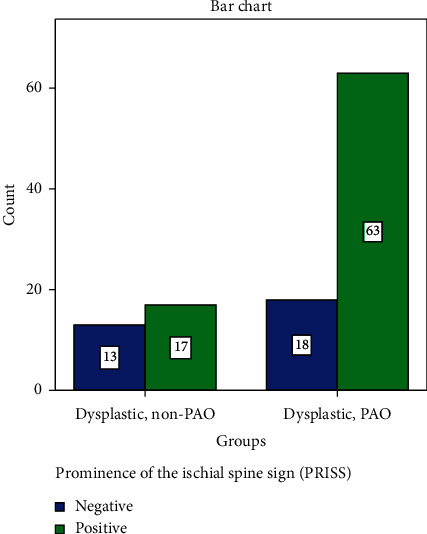
The crossover sign in all dysplastic hips (PAO vs. non-PAO); *χ*^2^ = .027, *P*=0.488.

We compared the radiographic measurements between operated and nonoperated hips. We found no significant difference in the ISI (Table 5). However, when taken as a binary sign, we found a significant association of the PRISS with the operated hips (Figure 4), whereas when assessed by the COS, there was no significant association (Figure 5).

## 4. Discussion

Due to the irregular nature of acetabular walls in dysplastic hips, these walls might provide unclear morphological data of acetabular version. We showed that the use of the ischial spine as a surrogate sign for acetabular retroversion is a valid method and that it can reflect general acetabular orientation.

In this study, when PRIS was positive, not only did it show that in hip dysplasia the ischial spine was able to reflect retroversion but its degree of pelvic protrusion (ischial spine index) was able to classify the acetabular version (Table 1 and Figure 3).

Additionally, the simultaneous presence of the COS, PWS, and PRISS signs returned the highest degree of retroversion index (Table 4). Consequently, this reflects a higher degree of acetabular retroversion. These 3 signs may indicate the involvement of the whole midsegment of the pelvis composed of the whole acetabulum and the ischial spine in the setting of retroversion. These hips were associated with an average of 33.6% retroversion index, the most pronounced among all groups tested. No studies were able to correlate the length of the ischial spine to measure the degree of acetabular version.

### 4.1. Ischial Spine Sign in Operated Hips

**Figure 6:**
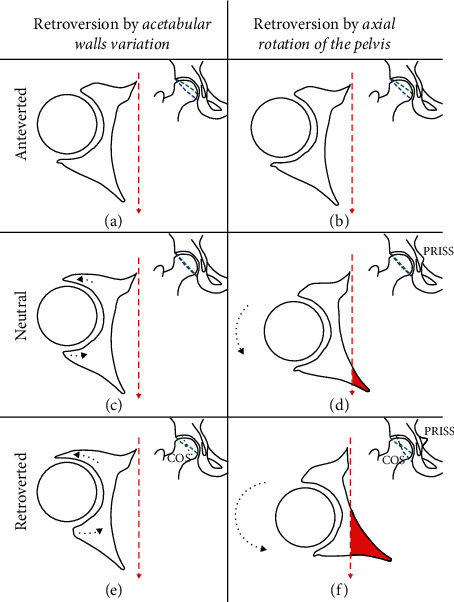
Simplified hip illustrations explaining the possible mechanisms of retroversion: acetabular walls variation versus axial rotation of the pelvis. The arrow depicts the direction of AP pelvic radiographs projection. The hip at the top right is a simulation of an AP pelvic X-ray showing the COS and PRISS for each corresponding setting where the anterior and posterior (green and blue dashed lines, respectively) acetabular walls are outlined. COS = crossover sign; PRISS = prominence of the ischial spine sign.

It was previously suggested that the presence of acetabular retroversion is probably independent of congenital hip dysplasia and that it appears to be a secondary factor in the appearance of acetabular dysplasia symptoms [12]. In that former study, retroversion was assessed using the retroversion index derived from the COS. The corresponding hips were tagged as retroverted based solely on the presence of the COS. Consequently, hips that are actually anatomically different (i.e., hips with the COS having a positive (Figure 6(f)) or negative PRISS (Figure 6(e)) were studied as one group, and no significant association was found between the COS and the requirement for PAO (Figure 5).

These seemingly identically retroverted hips (positive COS on AP radiograph) can be further subcategorized according to the PRISS, where in our current study, we filtered the retroverted dysplastic group according to this sign and showed that the dysplastic group requiring PAO (i.e. patient with symptomatic hip dysplasia) was, in addition to being more dysplastic, significantly associated with the PRISS (Figure 4). This hints that in the setting of hip dysplasia, acetabular retroversion might be involved in the manifestation of hip symptoms leading to PAO.

In order to interpret our results, we have to consider the midsegment of the pelvis including both the acetabulum and the ischial spine as a whole unit. In that setting, whenever you have a positive ischial spine sign protruding beyond the pelvic brim in the inner pelvis, it reflects an external rotation of this midsegment (Figure 6(f)).

Therefore, in this setting of a positive PRISS, the presence of a retroverted acetabulum with a positive PRISS could be explained by the concept of “combined retroversion” including first, the classic Reynolds theory where retroversion is caused by an overhang of the anterior wall or an underdeveloped posterior wall both producing a crossover sign on AP X-ray and occasionally causing pincer-type impingement. And second, the associated external rotation of the whole mid-pelvic segment exaggerating the retroversion and protruding the ischial spine internally beyond the pelvic brim.

We can conclude that surgical retroverted dysplastic hips were more dysplastic and had a more pronounced combined retroversion as assessed by the association of a positive PRISS. It suggests that retroversion does contribute to the presence of symptoms but only as long as the retroversion is associated with axial rotation of the hemipelvis.

For the surgeon dealing with PAO surgery, the presence of the positive PRISS should trigger caution that this hip might be more dysplastic and more symptomatic and hence more surgical. And the surgeon should also have the notion of combined retroversion in mind, while reorienting the osteotomized fragment.

To note, the lack of significant association of the length of the ischial spine does not reflect contradicting results when comparing the PAO group to the non-PAO one. Ultimately, the surgeon relies on the presence or absence of the studied ischial spine sign radiographically to aid in the surgical decision making (rather than measuring its corresponding length that was shown here to lack any clinical significance).

## 5. Conclusions

The PRISS is a valid sign for diagnosing acetabular retroversion in dysplastic hips requiring corrective surgery. The findings in our study are important in guiding corrective osteotomy of the acetabulum. Additionally, ISI, the newly described ischial spine index, allows comprehensive assessment of the ischial spine taking into account variation in hip anatomy and ischial spine patient-specific morphology.

Lastly, the association of the PRISS with hips requiring PAO alludes to a considerable role of retroversion in symptomatic patients. The ischial spine conveys morphological information pertaining to acetabular retroversion that is otherwise lacking with the COS and PWS in the setting of surgical hip dysplasia.

## Figures and Tables

**Table 1:** Comparison of the 3 acetabular version groups where analysis of variance was performed.

Acetabular version	Ischial spine index (%)	PRIS 1 (mm)	PRIS 2 (mm)	Age (years)	CE angle (degrees)	CA angle (degrees)	Tönnis angle
Anteverted (*n* = 72)	5.64 ± 13.08 (0–63)	0.46 ± 1.17 (0–6.4)	6.29 ± 2.55 (2.40–18.1)	31.32 ± 10.87 (15–56)	5.31 ± 10.39 (-26–30)	-0.33 ± 14.44 (-44–28)	25.36 ± 6.70 (7–45)
Neutral (*n* = 34)	20.30 ± 24.68 (0–80)	1.73 ± 2.19 (0–7)	7.16 ± 2.33 (3–11.8)	33.06 ± 9.76 (15–48)	8.22 ± 9.30 (−17–24)	1.03 ± 14.96 (−30–32)	22.16 ± 5.82 (9–35)
Retroverted (*n* = 78)	34.16 ± 25.48 (0–80)	3.32 ± 3.09 (0–13.80)	8.47 ± 3.26 (3–17.8)	28.12 ± 10.04 (15–48)	4.28 ± 12.54 (−50–26)	−0.11 ± 19.52 (−46–47)	24.11 ± 8.04 (0–44)
Total (*n* = 184)	21.33 ± 24.83 (0–80)	1.90 ± 2.66 (0–13.8)	7.38 ± 2.99 (2.4–18.1)	30.29 ± 10.45 (15–56)	5.42 ± 11.21 (−50–30)	0.02 ± 16.76 (−46–57)	24.23 ± 7.21 (0–45)
*F*	33.665	27.94	11.1	3.44	1.55	0.07	2.43
Mean square	15299.248	153.843	90.380	367.355	194.053	20.993	124.857
*P*	<0.001	<0.001	<0.001	0.03	0.21	0.92	0.09

All values are described as mean ± SD (range). CE angle = Wiberg lateral coverage angle; Tönnis angle = acetabular bearing surface index; CA angle = Lequesne anterior coverage angle.

**Table 2:** Correlations of coxometric measurements with length of ischial spine.

		PRIS 1		PRIS 2		ISI	
		*r* coefficient	*P*	*r* coefficient	*P*	*r* coefficient	*P*
CD		0.612^*∗∗*^	<0.001	0.466^*∗∗*^	<0.001	0.556^*∗∗*^	<0.001
RI		0.416^*∗∗*^	0.003	0.154	0.221	0.294^*∗∗*^	0.009

PRIS = prominence of the ischial spine; ISI = ischial spine index; CD = crossover distance; RI = retroversion index.

**Table 3:** Comparison of coxometric measurements according to ischial spine sign.

	Ischial spine		Mean difference (%)	*P*
	Present (%)	Absent (%)		
RI	33.37	31.60	1.76	0.593
CD	11.45	2.48	8.97	<0.001

RI = retroversion index; CD = crossover distance.

**Table 4:** Retroversion index according to radiographic markers (PWS, COS, and ISS).

	Retroversion index	
	PRISS (+) (%)	PRISS (−) (%)
(+) COS, (+) PWS	33.6%	29.9
(+) COS, (−) PWS	25.6%	23.8

**Table 5:** Comparison of the dysplastic PAO vs. dysplastic non-PAO groups.

Dysplastic hips	ISI (%)	PRIS 1 (mm)	PRIS 2 (mm)	RI (%)	Age (years)	CE angle (degrees)	CA angle (degrees)	Tönnis angle (degrees)
Operated hips (*n* = 192)	20.51 ± 24.77 (0–80)	1.91 ± 2.66 (0–6.4)	7.38 ± 2.99 (2.40–18.1)	32.97 ± 12.25 (10.2–64.3)	30.32 ± 10.87 (15–56)	5.45 ± 11.19 (−50–30)	6.59 ± 16.19 (−46–47)	24.19 ± 7.21 (0–45)
Nonoperated hips (*n* = 80)	16.05 ± 22.59 (0–81)	1.35 ± 2.01 (0–7)	7.06 ± 2.87 (2.3–20)	28.64 ± 9.24 (17.8–51.2)	30.75 ± 10.43 (15–56)	15.43 ± 8.14 (−10–28)	0.02 ± 16.71 (−25–37)	17.76 ± 5.95 (5–37)
Total (*n* = 272)	19.18 ± 24.18 (0–81)	1.90 ± 2.66 (0–13.8)	7.38 ± 2.99 (2.3–20)	31.8 ± 11.64 (10.2–64.3)	30.44 ± 10.53 (15–56)	8.39 ± 11.33 (−50–30)	0.02 ± 16.76 (−46–47)	22.30 ± 7.45 (0–45)
*P*	0.171	0.03	0.449	0.081	0.761	<0.001	0.059	<0.001

All values are described as mean ± SD (range). ISI = ischial spine index; RI = retroversion index.

## Data Availability

The data used to support the findings of this study are available from the corresponding author upon request.
